# Adaptive traits for chitin utilization in the saprotrophic aquatic chytrid fungus *Rhizoclosmatium globosum*

**DOI:** 10.1098/rspb.2025.0337

**Published:** 2025-05-28

**Authors:** Nathan Chrismas, Kimberley Bird, Davis Laundon, Chloe Lieng, Poppy Hesketh-Best, Michael Cunliffe

**Affiliations:** ^1^ The Marine Biological Association, Plymouth, Devon PL1 2PB, UK; ^2^ School of Biological and Marine Sciences, University of Plymouth, Plymouth, Devon PL4 8AA, UK

**Keywords:** chytrids, Chytridiomycota, aquatic, horizontal gene transfer, gene expansion

## Abstract

The Chytridiomycota (chytrids) are early diverging fungi, many of which function in ecosystems as saprotrophs; however, associated adaptive traits are poorly understood. We focused on chitin degradation, a common ecosystem function of aquatic chytrids, using the model chitinophilic *Rhizoclosmatium globosum* and comparison of other chytrid genomes. Zoospores are chemotactic to the chitin monomer *N-*acetylglucosamine and accelerate development when grown with chitin. The *R. globosum* secretome is dominated by different glycoside hydrolase (GH) family GH18 chitinases, with abundance matching reciprocal transcriptome mRNA sequences. Models of the secreted chitinases indicate a range of sizes and domain configurations. Along with *R. globosum*, the genomes of other chitinophilic chytrids also have expanded inventories of GH-encoding genes responsible for chitin processing. Several *R. globosum* GH18 chitinases have bacteria-like chitin-binding module domains, also present in the genomes of other chitinophilic chytrids yet absent in non-chitinophilic chytrids. Chemotaxis, increased abundance and diversity of secreted chitinases, complemented with the acquisition of novel chitin-binding capability, are probably adaptive traits that facilitate chitin saprotrophy. Our study reveals the underpinning mechanisms that have supported the niche expansion of some chytrids to utilize lucrative chitin-rich particles in aquatic ecosystems and is a demonstration of the adaptive ability of this successful fungal group.

## 1. Introduction

Fungi are leading drivers of ecosystem processes, including the degradation of high-molecular weight, often recalcitrant and complex, biogenic organic polymers. As high-molecular weight material is too large to be absorbed across fungal cell walls, substrates must first be degraded in the extracellular environment. This process is carried out by enzymes, predominantly carbohydrate-active enzyme (CAZymes), secreted by the fungus as part of their secretome into the environment before subsequent smaller breakdown products are absorbed via osmotrophy [1]. Our understanding of the mechanisms underpinning fungal saprotrophy, including the importance of CAZymes, is largely based on decomposition of plant-based material by terrestrial Ascomycota and Basidiomycota, the evolution of which is synonymous with the successful expansion of the kingdom through exploitation of diverse ecological niches [2–4].

**Figure 1.**
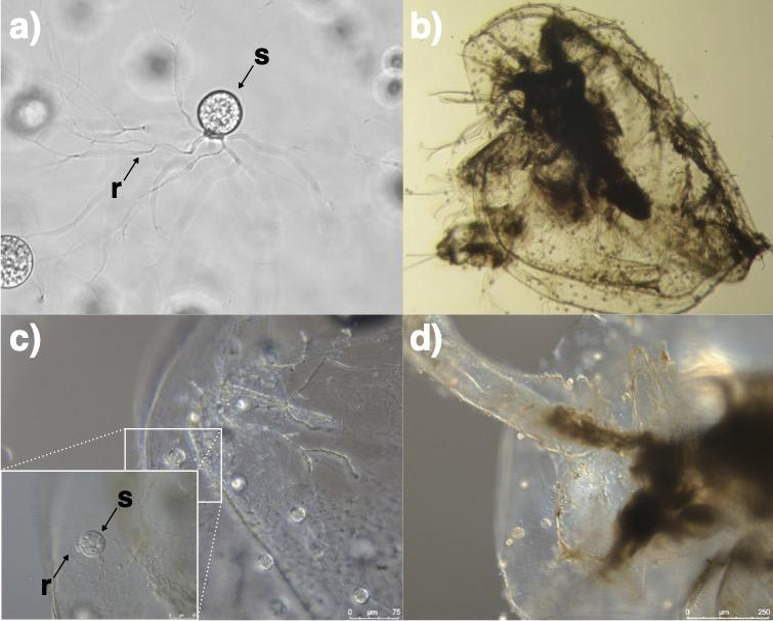
*Rhizoclosmatium globosum* JEL800 with branching rhizoids (r) used for attaching to substrates and sporangium (s) inside which develop multiple swimming zoospores. (b–d) *Rhizoclosmatium globosum* JEL800 attached to *Daphnia* exoskeleton across various levels of magnification. Note rhizoids (*r*) penetrating the exoskeleton surface.

The Chytridiomycota (chytrids) is a group of early-diverging fungi that are typically unicellular, developing into a sporangium with substrate-attaching and feeding rhizoids (figure 1a). Within mature sporangia (zoosporangia), multiple uniflagellate zoospores form that lack a cell wall and are usually motile when released into the environment. Once a suitable substrate is found by swimming zoospores they attach and encyst, developing into a new sporangium with rhizoids to repeat the life cycle [5]. Chytrids broadly occupy two major functional roles in ecosystems, either as (i) parasites, mainly of phytoplankton with some exceptions including amphibian specialists or (ii) saprotrophs that degrade recalcitrant organic biopolymers. Multiple independent transitions between parasitism and saprotrophy have occurred across the chytrids, with saprotrophy likely a derived trait and the ancestral trait being phytoplankton parasitism [6]. The mechanisms underpinning aquatic chytrid saprotrophy, including development of adaptive functional traits, are currently poorly understood [7],

Chitin-based particles such as arthropod exoskeleton remains (exuviae) are prominent aquatic saprotrophic chytrid habitats [8] (figure 1b–d). Chitin is a polymer of *N*-acetylglucosamine (GlcNAc) and is the second most abundant polysaccharide on Earth after cellulose. The aims of this study were to determine the mechanisms underpinning chytrid chitin saprotrophy by focusing on two specific questions: (i) what chitin-associated behaviours are shown by swimming zoospores and (ii) what is the CAZyme machinery of the secretome involved in chitin saprotrophy (i.e. secretome CAZymes and associated genes/mRNA). Our approach was to use the model saprotroph *Rhizoclosmatium globosum* JEL800 to determine new insights into chytrid ecology. *Rhizoclosmatium globosum* is an aquatic chytrid found prevalent in nature attached to chitin-rich arthropod exuviae [8]. The strain *R. globosum* JEL800 is an emerging model for studies of chytrid fungi [9–13] that was isolated by chitin baiting (Joyce E. Longcore, 2016, personal communication) and grows readily on chitin as a sole carbon source [11].

## 2. Material and methods

### (a) *Rhizoclosmatium globosum* JEL800 maintenance, growth and microscopy

*Rhizoclosmatium globosum* JEL800 was routinely maintained on peptidised milk, tryptone and glucose (PmTG) agar plates [14] at 22°C in the dark. For experimental analysis, *R. globosum* JEL800 was also grown with modified Bold’s Basal Medium (BBM) [11] diluted 10-fold and with the addition of 0.25 g ammonium sulfate and 0.5 ml f/2 vitamin stock in 1 l [15]. Samples for transcriptome and secretome analysis were produced from the same cultures (*n* = 4). *Rhizoclosmatium globosum* JEL800 was grown in 50 ml BBM with 10 mM chitin (powder, from shrimp shells, Sigma-Aldrich) in 250 ml Erlenmeyer flasks inoculated with approximately 14 000 zoospores and shaking at 70 rpm. Cultures were harvested after 7 d growth by transferring contents to a 50 ml centrifuge tube and centrifuging at 4000*g* for 10 min. Cell pellets were snap frozen with liquid nitrogen, stored at –80°C and used for transcriptome analysis. The supernatant was removed and Halt Protease & Phosphate Inhibitor Cocktail (Thermo) added at 5 µl 50 ml^−1^ with mixing by inversion. Protease treated supernatant (approx. 50 ml) was concentrated to approximately 250 µl in 15 ml batches at 4000 G for 20 min each (total approx. 1 h) using 10 kDa Amicon Ultra 15 ml Centrifugal Filters (Millipore). Concentrated supernatant was transferred to a 3 kDa Pierce Protein Concentrator PES 0.5 ml (Thermo) and centrifuged for 20 min at 15 000*g* to a final volume of approximately 100 µl. All centrifuge steps were carried out at 4°C and samples stored at −80°C. Comparative proteomics of the replicate (*n* = 4) secretome samples was run with tandem mass tagging (TMT) [16] using TMTpro reagents (TMTpro Thermo Fisher) and nano-liquid chromatography tandem mass spectrometry using an Orbitrap Fusion Lumos mass spectrometer (Thermo Scientific) at the University of Bristol Proteomics Facility (see electronic supplementary material, methods). Proteome data analysis was conducted with searches against the *R. globosum* JEL800 UniProt database using Proteome Discover software (Thermo Fisher). Reciprocal identified secretome proteins and mRNA sequences from the cellular transcriptome (see below) where manually cross-referenced with the corresponding genes in the *R. globosum* JEL800 reference genome.

To screen a range of alternative non-chitin carbon sources used by *R. globosum* JEL800 for growth, mature cultures on PmTG agar plates were sporulated using 4 ml dH_2_O for approximately 2 h at room temperature. Zoospores were then harvested and passed through a 10 µm sieved before being washed 3 times in a carbon-free BBM by centrifuging at 4000 G for 10 min at 4°C and re-suspended in a carbon-free BBM. Biolog PM 1 and PM 2A Carbon Utilisation Assay plates (Biolog Inc.) were inoculated with 150 µl per well at a final density of 1 × 10^3^ zoospores per well. Assay plates were incubated at 22°C, and absorbance at 405 nm (OD_405_) was measured periodically using a plate reader (CLARIOstar).

To demonstrate *R. globosum* JEL800 growth on an arthropod exoskeleton, *Daphnia* were acquired from a local aquarium supplier and cleaned by incubation in 10% sodium dodecyl sulphate solution for 30 min, followed by 100% ethanol for 1 h and then autoclaved in dH_2_O. Carapaces were washed twice in sterile dH_2_O between each step. Culture dishes containing 3 ml BBM and 1 carapace were inoculated with 500 µl zoospore suspension (1.03 × 10^6^ zoospores ml^−1^) and incubated at 22°C. Chytrids growing on carapaces were imaged with differential interference contrast using a Leica Dmi8 microscope (Leica, Germany).

### (b) *Rhizoclosmatium globosum* JEL800 zoospore chemotaxis and motility

Prior to chemotaxis and motility experiments, *R. globosum* JEL800 was maintained on agar plates made with PmTG, or BBM with 5 mM GlcNAc or chitin for at least three generations. Mature plates were sporulated and 1 ml zoospores spread onto fresh media and dried to create a lawn. Lawns were incubated at 22°C for 24 h (PmTG and GlcNAc) and 48 h (chitin) to allow zoosporangia to develop and mature. For chemotaxis experiments, mature lawns were sporulated with 5 ml sterile dH_2_O, and after 30 min zoospores were harvested and passed through a 10 µm sieve, enumerated using a LUNA Automated Cell Counter (Logos Biosystems) and motility checked using a haemocytometer. Only zoospore suspensions with motility >80% and approximately 1 × 10^7^ cells ml^−1^ were used for experiments. A total of 2 ml zoospore suspension was placed in a 50 ml centrifuge tube and the top sealed with parafilm. Syringes (1 ml) with 21G needles containing either 150 µl 10 mM GlcNAc, 10 mM glucose or dH_2_O only (control) were held in the zoospore suspension and incubated at 22°C in the dark. After 1 h, the contents of the syringe were emptied into a 1.5 ml tube and fixed with glutaraldehyde (final concentration of 0.5 %) and zoospores enumerated using a haemocytometer. Chemotaxis was determined if a significantly greater number of zoospores was present in the potential chemoattract versus the H_2_O only control. Following a Shapiro–Wilk test for normality, one way analysis of variance (ANOVA) with post hoc pairwise Tukey test was used to determine difference between chemotaxis substrate targets (SigmaPlot). For motility experiments, zoospore lawns were sporulated with 3 ml sterile dH_2_O, a 1 ml sample was taken immediately and a subsample of this was glutaraldehyde fixed to determine the starting concentration. Non-motile zoospores were enumerated using a haemocytometer every 5 min for 1 h and % motility was calculated from the starting concentration. Differences between treatments for the motility experiments were determined with a Mann–Whitney rank sum test (SigmaPlot).

### (c) RNA extraction and transcriptome analysis

RNA was extracted from the cell pellets (*n* = 4) using the RNeasy Micro kit (Qiagen) according to the manufacturer’s instructions. Pellets were thawed in 2 ml RLT lysis buffer (Qiagen) containing 10 μl ml^−1^ of 2-mercaptoethanol, incubated at room temperature for 5 min and mixed periodically using a vortex. Cell debris was removed by centrifuging at 4000 G for 1 min, the lysate was recovered through a QIA shredder (Qiagen). An equal volume of 100% ethanol was added to the homogenized lysate before being transferred in batches to the RNeasy extraction column including on column DNase digestion step using the RNase-Free DNase set (Qiagen). RNA was quantified using a NanoDrop 1000 spectrophotometer (Thermo) and the RNA BR assay kit (Invitrogen) on the Qubit 4 fluorometer (Invitrogen). RNA quality was assessed using the RNA 6000 Nano kit total RNA assay (Agilent) run on the 2100 Bioanalyzer instrument (Agilent).

Messenger RNA (mRNA) was purified from total RNA using poly-T oligo-attached magnetic beads and underwent library preparation at Novogene (see electronic supplementary material, methods). The library was further checked then subjected to Illumina sequencing. Raw data (raw reads) in fastq format were first processed through fastp software. Clean data (clean reads) were obtained by removing adapter reads, ploy-N and low-quality reads from raw data. Q20, Q30, guanine-cytosine (GC) content and the clean data were calculated (data quality control summary provided in electronic supplementary material, table S1). All the downstream analyses were based on the clean data. HISAT2 was used to map the paired-end clean reads to the *R. globosum* JEL800 reference genome (mapping results are provided in electronic supplementary material, table S1). FeatureCounts (2.0.6) was used to count the read numbers mapped to each gene and from which Fragments Per Kilobase of transcript per million mapped reads (FPKM) was calculated.

### (d) Protein modelling from gene sequences

GH18 and GH20 models were retrieved from the AlphaFold Protein Structure Database [17,18]. Total model volumes were predicted using VADAR 1.8 [19], and binding site volumes were predicted using CASTp 3.0 [20] with default settings. The complete model for ORY35945 including the GH18 domain, linker and CBM5 domain was visualized in UCSF Chimera [21]. Graphics were edited using Inkscape (http://inkscape.org).

### (e) Comparison across Chytridiomycota genomes

The counts of predicted genes were retrieved from Chytridiomycota reference genomes from Mycocosm (https://mycocosm.jgi.doe.gov/, accessed October 2024) [22] (electronic supplementary material, table S2). Conserved domains in *R. globosum* JEL800 GH18 and GH20 genes were identified using conserved domain (CD)-search [23] and aligned separately in MAFFT [24] using E-INS-i before maximum-likelihood phylogenetic analysis was carried out using the CIPRES [25] implementation of RaxML v. 8.2.10 [26] using the CAT model and the BLOSUM62 matrix for divergent protein sequences with automatic bootstrapping. Domain trees were visualized using FigTree v. 1.4.4 (http://tree.bio.ed.ac.uk/software/figtree/). GH18 domains were further subclassified by comparison with a phylum-wide sampling of fungal GH18 domains [27] (electronic supplementary material, figure S1). The presence of signal peptides in each gene was predicted using the SignalP 4.1 webserver [28]. Complete protein domains were plotted using genoplotR [29].

### (f) *Rhizoclosmatium globosum* JEL800 CBM5 horizontal gene transfer identification

A combination of BLAST similarity searches and phylogenetic analysis was used to identify potential horizontal gene transfer (HGT) events. BLASTP searches were performed with the *R. globosum* JEL800 CBM5 amino acid (aa) sequences with hits downloaded to a combined fasta file. All CBM5 aa sequences were aligned using the maximum-likelihood method based on the JTT matrix-based model (MEGA). CBM5 aa sequences from non-chytrid fungi were added manually, and the CBM5 aa sequence from the archaeon *Haloferax mediterranei* was used as the outgroup.

## 3. Results

### (a) *Z*oospore swimming behaviours indicate adaptations to chitinophilic lifestyle

**Figure 2.**
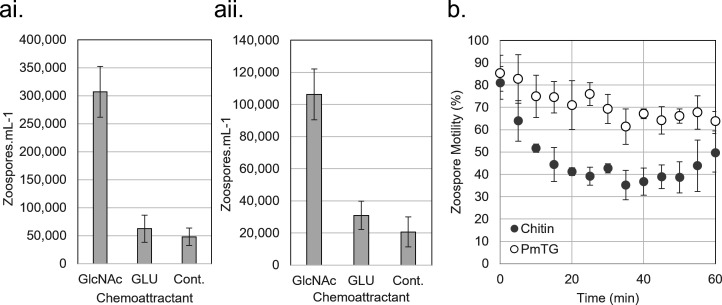
Chemotactic response of *Rhizoclosmatium globosum* JEL800 zoospores to the chitin monomer *N*-acetylglucosamine (GlcNAc) compared with glucose (GLU) and no-attractant control (Cont.) from cultures grown with (ai) GlcNAc as the sole carbon source and (aii) in complex GlcNAc-free medium PmTG. (b) Loss of motility in *R. globosum* JEL800 zoospores from cultures grown with chitin as the sole carbon source compared with the complex medium PmTG.

Swimming *R. globosum* JEL800 zoospores are chemotactic towards the chitin monomer GlcNAc. Targeted swimming toward GlcNAc occurred with zoospores from cultures grown with GlcNAc as the carbon source (ANOVA/Tukey *p* < 0.001) (figure 2ai) and when grown without GlcNAc in the complex medium PmTG (ANOVA/Tukey *p* < 0.001) (figure 2aii). Even though *R. globosum* grows with glucose as an alternative carbon source (electronic supplementary material, figure S2), chemotaxis did not occur towards glucose with either GlcNAc or PmTG grown cultures (GlcNAc ANOVA/Tukey p 0.794; PmTG ANOVA/Tukey p 0.461) (figure 2ai,aii).

We made several attempts to determine chemotaxis in zoospores from cultures grown with chitin but were unable to conduct the assay due to apparent reduction in zoospore motility. To explore this further, we compared general motility (i.e. swimming versus stationary) of zoospores harvested from cultures grown with chitin to the motility of zoospores from cultures grown with PmTG, showing that chitin-grown zoospores rapidly lose motility after release compared with zoospores grown with PmTG which remained motile for longer (Mann–Whitney rank sum test *p* < 0.001; figure 2b).

### (b) *Rhizoclosmatium globosum* chitin secretome CAZymes

**Figure 3.**
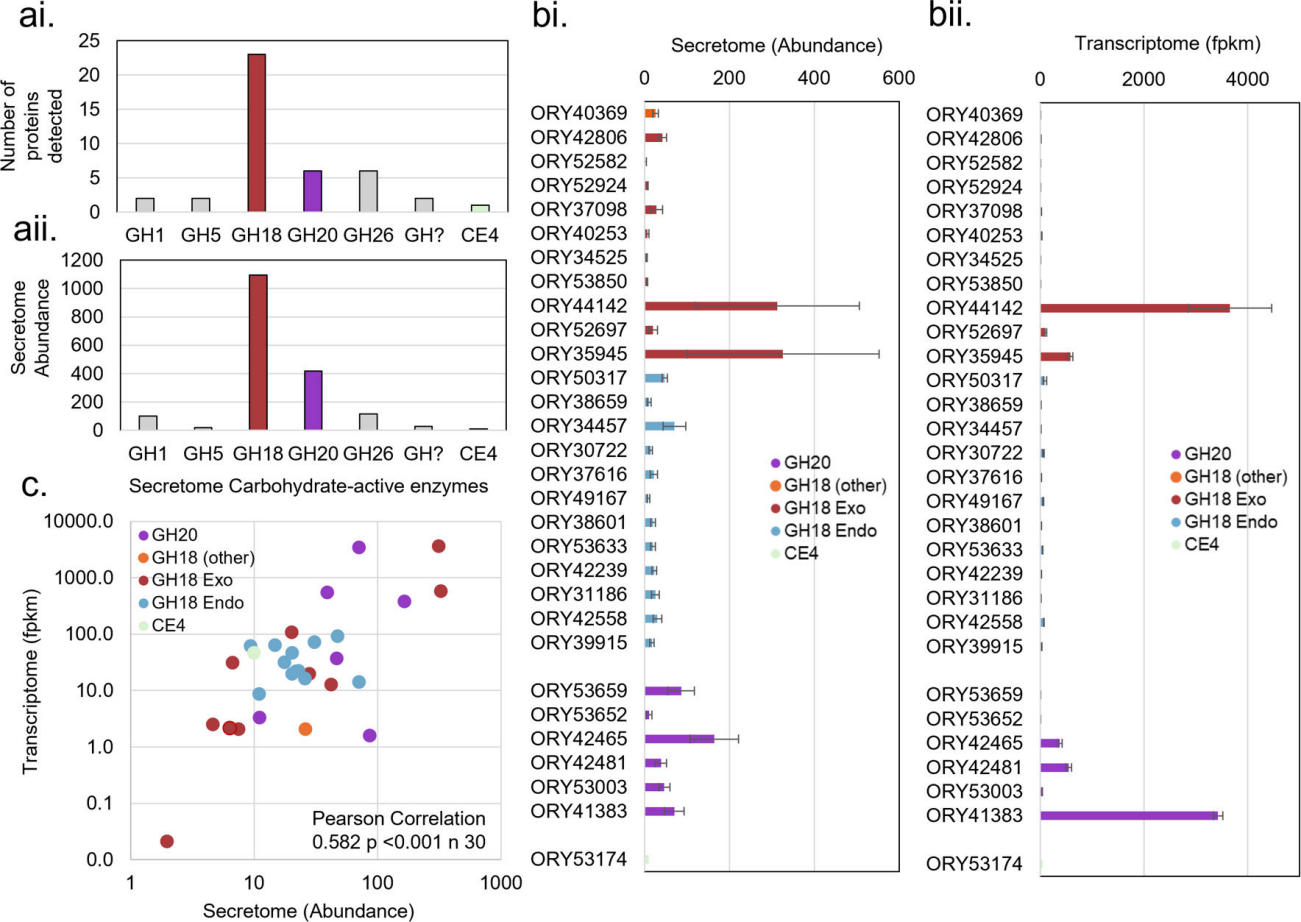
Number (ai) and abundance (aii) of carbohydrate-active enzymes (CAZymes) in the secretome of *Rhizoclosmatium globosum* JEL800 grown with chitin. (bi) Secretome abundance and (bii) transcriptome abundance (mRNA FPKM) for all individual CAZymes present in the secretome potentially involved with chitin degradation. (c) Relationship of abundance of proteins in the secretome potentially involved with chitin degradation to equivalent mRNA in the transcriptome.

**Figure 4.**
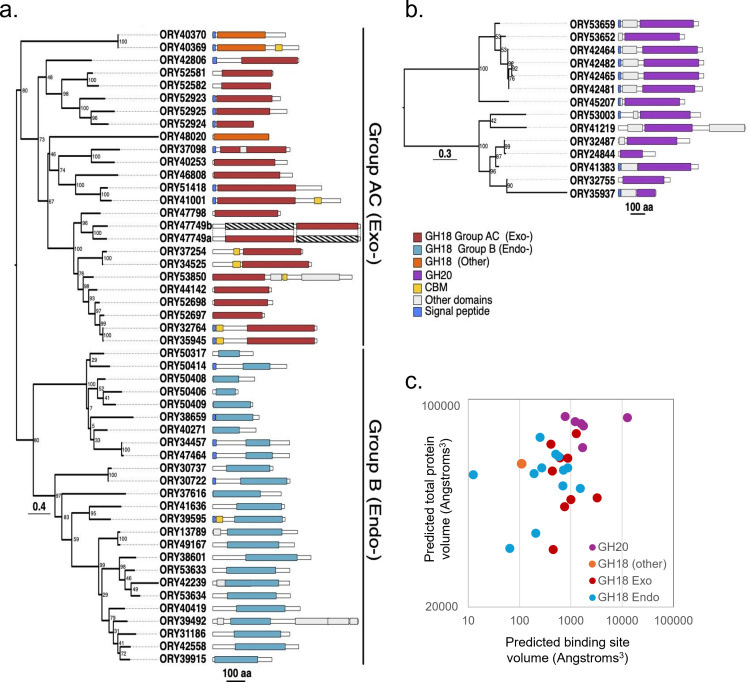
Maximum-likelihood phylogenies of *Rhizoclosmatium globosum* JEL800 (a) GH18 and (b) GH20 domains showing architecture of GH domain-containing genes. GH18 genes encode for proteins with either endo-(Group B) or exochitinase (Group AC) activity (electronic supplementary material, figure S1). (c) Relationship of substrate binding site volume to total protein volume of secretome proteins based on GH18 and GH20 models retrieved from the AlphaFold Protein Structure Database. Total model volumes predicted using VADAR 1.8 [19], and binding site volumes were predicted using CASTp 3.0 [20].

The CAZymes in the *R. globosum* JEL800 secretome were dominated by glycoside hydrolase family 18 (GH18) chitinases, making up 64.5% of the different CAZymes determined (figure 3ai) and 61.5% CAZyme abundance (figure 3aii). A total of 23 proteins from GH18 domain-containing genes were detected, with 10 from Group AC exochitinases (cleaving terminal ends of GlcNAc polymers), 12 from Group B endochitinases (introducing random breaks into chitin) and 1 other (figure 3bi). The secretome GH18 proteins accounted for almost half (47%) of the GH18 genes in the *R. globosum* JEL800 genome (figure 4a). The glycoside hydrolase family 20 (GH20) β-*N*-acetyl-d-hexosaminidases (cleaving chitin oligosaccharides) was the second major secretome CAZyme group, making up 14.3% of different CAZymes proteins (figure 3ai) and 23.4% CAZymes abundance (figure 3aii). All GH18 and GH20 secretome proteins (figure 3bi) matched reciprocal mRNA sequences detected in the cellular transcriptome (figure 3bii), with a positive correlation of secretome protein abundance with mRNA FPKM (Pearson correlation 0.582, *p* < 0.001 and *n =* 30) (figure 3c).

Other CAZymes potentially involved in chitin degradation were present at lower numbers and abundances in the *R. globosum* JEL800 secretome compared with GH18 and GH20 proteins (figure 3ai,aii). A GH19 chitinase was detected in the secretome that is part of the same open reading frame as one of the 23 GH18 proteins detected (ORY53850). Also present in the secretome was a carbohydrate esterase family 4 (CE4) chitin deacetylase. Other minor CAZymes with no known functional role in chitin degradation were also present along with two glycoside hydrolases that could not be further identified (figure 3ai,aii).

### (c) *Rhizoclosmatium globosum* GH18 and GH20 diversity and domain architecture

Given the prominence of GH18 and GH20 proteins in the *R. globosum* JEL800 chitin secretome they were examined more closely at the gene/genome level. Forty-nine *R. globosum* JEL800 genes contain GH18 domains (figure 4a). Maximum-likelihood phylogenetic analysis of the GH18 domains revealed two major clades that corresponded with either Group AC (predominantly exochitinases) or Group B (predominantly endochitinases) (figure 4a). Group AC contained domains for 24 proteins, with 21 forming a large sub-clade, with two domains appearing in ORY47749. Although ORY48020 fell within this clade, it was separated by a long branch with relatively low bootstrap support. ORY40370 and ORY40369 fell outside the main clade. Group B contained domains from 25 proteins. One clade contained domains from 16 proteins, and a second clade contained a variety of GH18 domains from 9 proteins with low bootstrap support for several branches within this clade. Fourteen *R. globosum* JEL800 genes were found to contain GH20 domains (figure 4b), Maximum-likelihood phylogenetic analysis of which revealed two well-supported clades, each containing seven genes, nine of which had associated signal peptides. Predicted structures of the proteins encoded from GH18 domain-containing genes that were detected as secretome proteins suggest that *R. globosum* JEL800 chitinases are variable both in terms of total size and substrate binding site size (figure 4c).

### (d) Comparison of chitin degradation CAZyme encoding genes in *Rhizoclosmatium globosum* and other Chytridiomycota

**Figure 5.**
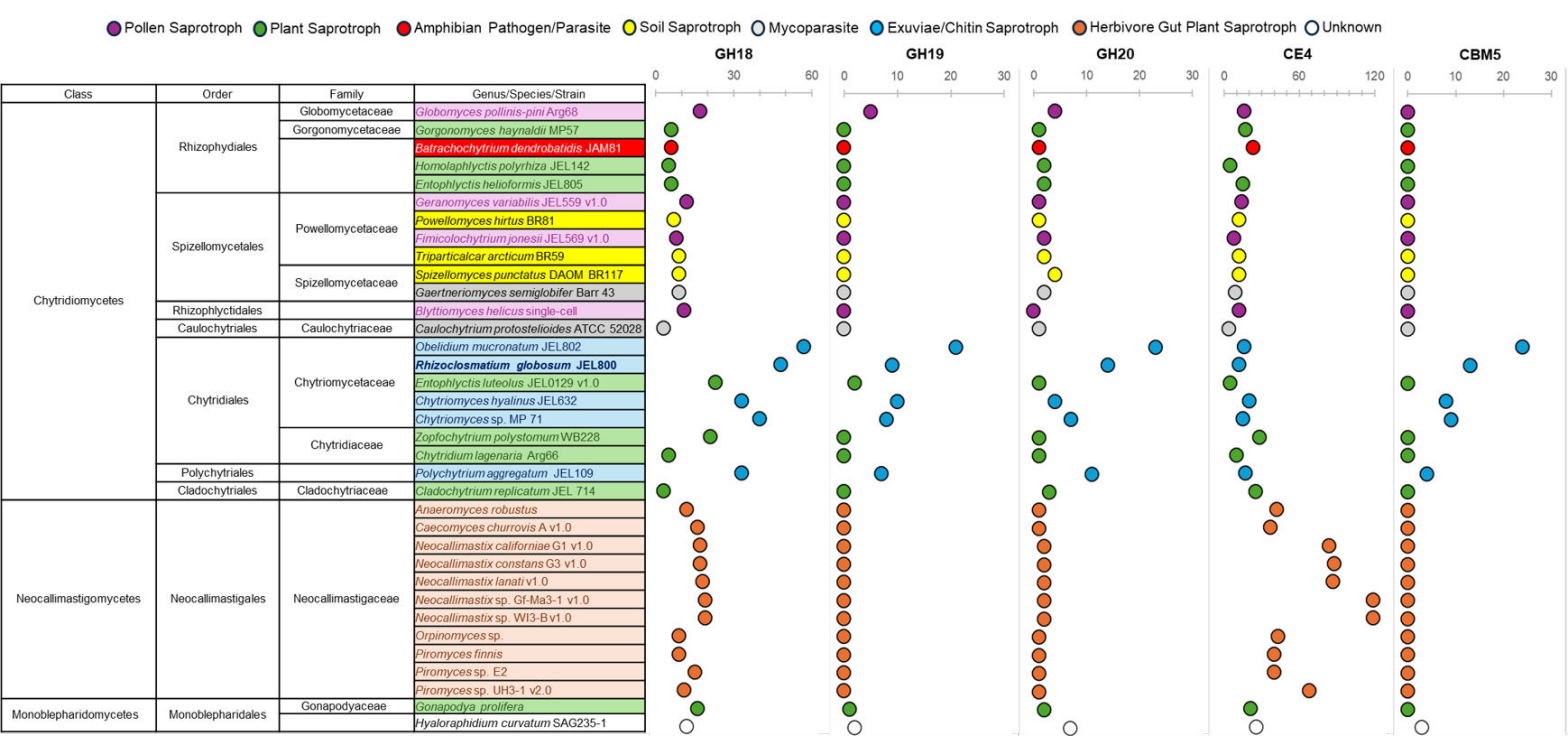
Comparison of the abundance of glycoside hydrolase GH18 and GH19 chitinase, GH20 β-*N*-acetyl-d-hexosaminidase, carbohydrate esterase CE4 chitin deacetylase and carbohydrate binding module CBM5 encoding genes in the genomes of *Rhizoclosmatium globosum* JEL800 and 34 other members of the Chytridiomycota that occupy diverse ecological niches (electronic supplementary material, table S2).

The abundance of GH18 and GH20 encoding genes in the *R. globosum* JEL800 genome was compared with gene abundance in the reference genomes of 34 other chytrids across the Chytridiomycota that occupy the same niche (i.e. other chitin-rich arthropod exuviae saprotrophs) and also a range of other niches (e.g. pollen saprotrophs, amphibian pathogen and herbivore gut plant material saprotrophs) (figure 5; electronic supplementary material, table S2). Chytrids associated with chitin-rich exuviae in nature and isolated by chitin-baiting belong to the Chytridiales family Chytriomycetaceae except *Polychytrium aggregatum* JEL109 from the order Polychytriales (figure 5). All the chitinophilic chytrid genomes showed the highest enrichments of GH18 encoding genes across the Chytridiomycota genomes surveyed (figure 5). All other chytrids (i.e. those occupying non-chitin associated niches) had relatively lower GH18 gene abundances than chitinophilic chytrids. A similar pattern of increased gene abundance in chitinophilic chytrids was observed for GH20 encoding genes (figure 5).

In terms of the alternative fungal chitinase family GH19, which was only detected as a minor component of the *R. globosum* JEL800 chitin secretome, GH19 encoding genes were also prevalent across the chitinophilic chytrid genomes and largely absent from the genomes of non-chitinophilic chytrids (figure 5). In the *R. globosum* JEL800 genome, there are nine domains encoding GH19 chitinases, with only one with considerable activity in the cellular transcriptome (ORY40385) (electronic supplementary material, figure S3).

### (e) Chitin-binding module domains in *Rhizoclosmatium globosum* and other chytrids

**Figure 6.**
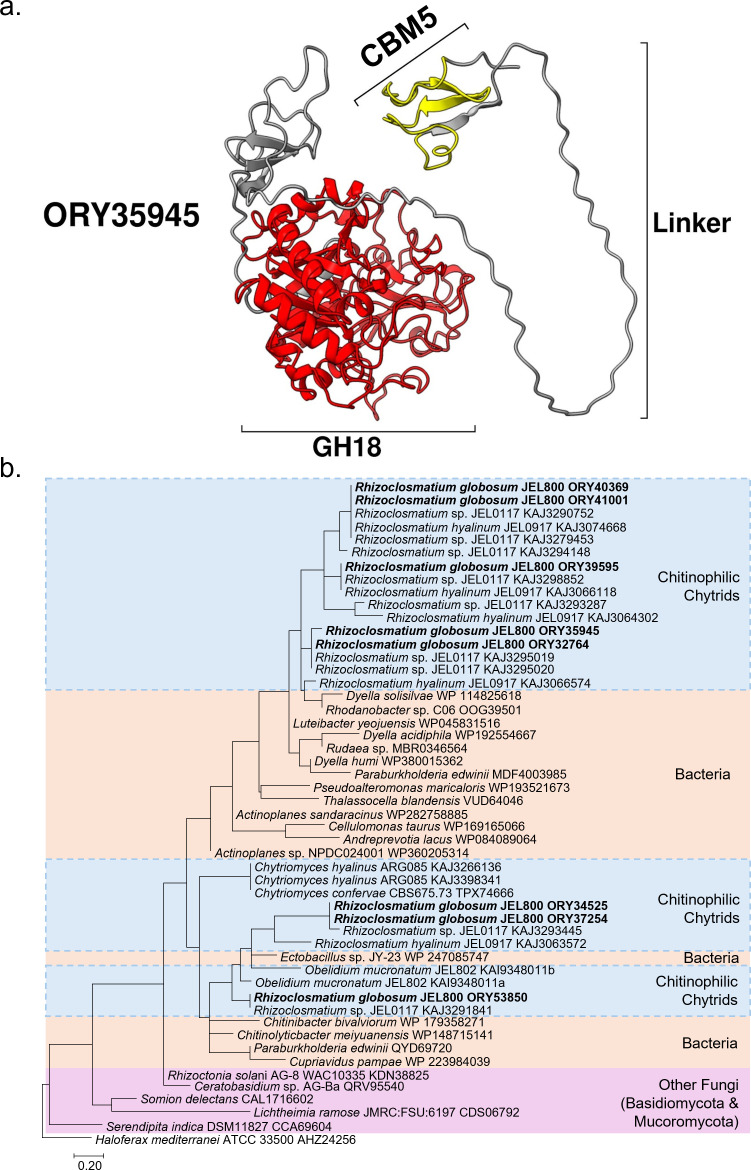
(a) AlphaFold predicted model of ORY35945. Colours are based on conserved domains: the carbohydrate binding module 5 (CBM5) domain (amino acid residues 22–36) is shown in yellow and the GH18 domain (amino acid residues 203–596) is shown in red. Both are joined by a linker region. Signal peptide region not shown. (b) Phylogenetic analysis by maximum-likelihood method showing affiliation of *Rhizoclosmatium globosum* JEL800 CBM5 amino acid sequences clustered with other chitinophilic chytrids and bacteria, including known chitinophilic bacteria, suggesting their bacterial origin. Other fungi (i.e. non chitinophilic chytrids) highlighted in pink. The CBM5 amino acid sequence from the archaeon *Haloferax mediterranei* is used as the outgroup.

Chitin-binding domains, all of which were carbohydrate-binding module 5 (CBM5)-type, were found with GH18 chitinases in the *R. globosum* JEL800 secretome. In terms of the genome, seven CBM5 domains were associated with Group AC and one with Group B GH18 chitinases (figure 4). Modelling of the secretome GH18 and associated CBM5 domains suggested variation in GH18–CBM5 domain configurations (electronic supplementary material, figure S4). For example, the abundant secretome GH18 chitinase ORY35945 predicted *C*-terminal GH18 domain and *N*-terminal CBM5 domain are joined by a linker domain (figure 6a), while comparative linker domains were absent in other GH18–CBM5 configurations (figure 4; electronic supplementary material, figure S4).

The abundance of genes containing CBM5 domains in the genomes of the chitinophilic chytrids was distinctive, with *R. globosum* JEL800 and *O. mucronatum* JEL802 particularly high (figure 5). Genes containing CBM5 domains were only detected in the genomes of chitinophilic chytrids except for the Monoblepharidomycete *Hyaloraphidium curvatum* SAG235-1.

Phylogenetic analysis of the *R. globosum* JEL800 CBM5 amino acid sequences associated with the GH18 chitinases showed that they nested with CBM5 sequences from other chitinophilic chytrids and bacteria (figure 6b). The closest other chitinophilic chytrids with similar CBM5 sequences were also chitinophilic *Rhizoclosmatium* isolates. Bacteria CBM5 sequences were from representatives from a range of phyla and included known chitinophilic species (e.g. *Chitinolyticbacter meiyuanensis* [30]). The eight *R. globosum* JEL800 CBM5 amino acid sequences were positioned separately in the phylogenetic analysis with other chitinophilic chytrids and bacteria.

## 4. Discussion

*Rhizoclosmatium globosum* JEL800 zoospores are chemotactic to the chitin monomer GlcNAc independent of the growth conditions of the previous generation, a trait that has obvious advantages for targeting chitin-rich substrates such as arthropod exuviae in complex aquatic environments. Previous laboratory-based experiments have demonstrated that some chytrid zoospores can selectively accumulate on chitin rather than cellulose implying that chemotaxis is taking place [31]. Chemotaxis has been shown in non-saprotrophic chytrid zoospores, particularly species that are parasites, with *Batrachochytrium dendrobatidis* zoospores chemotactic towards amphibian-produced compounds [32] and algal parasites targeting cell extracts of stressed hosts [33]. The mechanisms underpinning chemotaxis in aquatic chytrids are poorly understood [7]. It is possible that response to environmental signals could be linked to the rumposome, an enigmatic structure connecting the zoospore cell surface to the flagellum machinery that is present in *R. globosum* JEL800 zoospores [12] and is probably a site of calcium-mediated signalling [34]. Phototaxis has also been shown in chytrid zoospores, including with *R. globosum* JEL800, facilitated by a CyclOp-fusion protein-based sensory system [10]. With the marine alga-associated chytrid *Halomyces littoreus* (basionym *Rhizophydium littoreum* [35]), chemotaxis has been shown to be dependent on chemoattract concentration, with greater concentrations eliciting a stronger chemotactic response [36]. Together, this suggests that via chemotaxis and phototaxis, swimming chytrid zoospores utilize complex multi-signal mechanisms to navigate the aquatic environment that warrant future study.

The chemotactic response to GlcNAc shown here suggests that, in nature, chitinophilic chytrid zoospores respond to signals of ‘on-going’ chitin saprotophy that is releasing GlcNAc via degradation by other organisms. Chitin degradation in aquatic ecosystems is also performed by chitinophilic bacteria that share the same habitat and potential niche as chitinophilic chytrids such as *R. globosum* [37]. It is likely that a range of chytrid–bacteria interactions are associated with chitin-rich particles, including particle colonization, that involve the release of chemotactic signals from bacteria to chytrids and vice versa, depending on which are the primary or secondary colonizers [37].

We observed faster loss of motility in next generation zoospores when grown with chitin compared to non-chitin grown *R. globosum* JEL800. Accelerated transition from a non-feeding swimming zoospore to the substrate attaching stage of the life cycle (i.e. encystment) is a possible trait that could facilitate the rapid colonization and exploitation of ephemeral chitin-rich substrates such as mayfly exuviae in a dynamic resource-complex and competitive environment. Environmental controls on chytrid development including transition from the free-swimming zoospore stage are poorly understood [7]. In the frog pathogen *B. dendrobatidis*, host chemical cues (i.e. mucus) trigger encystment [38]. The chytrid zoospore behavioural traits that we report here that support chitin utilization are probably derived during zoosporogenesis and combine traits that are independent (i.e. GlcNAc chemotaxis) and dependent (i.e. accelerated zoospore-germling transition) of growth conditions (i.e. with or without chitin) of the previous generation.

The abundances of genes encoding chitinases and β-*N*-acetyl-d-hexosaminidases responsible for the breakdown of chitin and subsequent processing of chitin-derived GlcNAc were increased in chitinophilic chytrids relative to non-chitinolytic chytrids. It is important to reiterate that GH18 chitinases are probably also used for remodelling chitin in the chytrid cell wall based on analogous mechanisms in other fungi [39] and therefore necessary in all chytrids to some extent. Our study suggests that chitinase gene family expansion is varied within and between lineages, with an apparent increase in chitinase gene copy number associated with the chitinophilic members of the Chytridiales family Chytriomycetaceae and the Polychytriales chitinophilic *Polychytrium aggregatum*. Within the Chytriomycetaceae, the plant saprotroph *Entophlyctis luteolus* has apparently undergone gene reduction relative to the chitinophilic chytrids in the family. The relatedness of the orders Chytridiales and Polychytriales is not yet clear, with some comparative genomic studies suggesting they are more closely related [40] than others [6]. Future focused work is needed on gene expansion and loss across the Chytridiales and Polychytriales relative to niches occupied that could help elucidate their ancestral state(s) and evolutionary history.

Lineage-specific gene family expansion is a mechanism of adaptive evolution in eukaryotes and including in fungi [41]. Expansion of hydrolase gene families related to host/substrate specificity has been reported in other fungi. In non-chytrid fungi, necrotrophic Ascomycota and Basidiomycota exhibit expansion of GH28 family pectinases [42], and white rot fungi including *Armillaria* spp. (Basidiomycota) have an increased complement of genes encoding cellulose- and xylan-degrading enzymes [43]. Increased copy numbers of proteases have been associated with the evolution of amphibian pathogenicity in *B. dendrobatidis* by facilitating attachment and penetration of host cells [44,45]. In terms of GH18 gene expansion, as we show here in chitinophilic chytrids, similar pattern has been observed across other fungi including various Ascomycota [46] and ectomycorrhizal fungi [47].

A key feature of gene family expansion in fungi is that it facilitates exploitation of specific niches by allowing increased rates of enzyme production and functional novelty [48]. The increased chitin degradation inventory in chitinophilic chytrids may confer the ability to react quickly to substrate availability by transcribing multiple genes. Although cellular mRNA was detected for almost all the GH18 and GH20 genes encoded within the *R. globosum* JEL800 genome, not all transcribed genes were detected in the secretome. As well as accounting for cell wall processing cellular chitinases (which are yet to be characterized in chytrids), some of the untranslated mRNA may play a role in maintaining a bank of mRNA for a variety of chitinases, allowing *R. globosom* JEL800 to remain ‘primed’ for rapid protein synthesis.

Relatively few crystal structures of fungal chitinases have been characterized [49], and prediction of specific chitinase functions is therefore difficult [50] but variation in binding site volume relative to overall protein volume indicates structural diversity that may be related to variation in substrate, with different enzyme activities able to account for substrate complexity (discussed further below). The higher levels of GH18 Group AC exochitinases compared with Group B endochitinases in the chitin secretome suggest that cleavage of terminal GlcNAc from chitin is a particularly important characteristic of the chitinophilic lifestyle.

Alongside size complexity, we also show that some chitinases are multimodular with additional domains that potentially expand chitin degradation capability. Considering the major secreted chitinase ORY35945, this has broadly analogous domain configurations with the cellulose degrading endoglucanase in *Rhizopus stolonifer* (Mucoromycota) with a C-terminal catalytic domain linked with a N-terminal CBM [51]. The function of linkers between catalytic and binding domains has not been considered with chitinases but has been studied with cellulose- and lignocellulose-degrading enzymes and suggest that linkers may have multiple roles including general improvements in catalytic efficiency [51–53], promotion of localized and repeated substrate processing [52] and also have an additional substrate binding role [53]. Homologous CBM5 domains from the chitinophilic bacterium *Serratia marcescens* exhibit affinity specifically for α-chitin and promote chitin degradation in the presence of other polysaccharides [54]. In nature, chitinophilic chytrids are observed growing on arthropod exoskeleton remains [8] in which chitin is not available as a distinct molecule but instead packaged within a complex heterologous organic matrix. Even though exoskeleton matrices likely vary between source taxa, the general structure is based around crystalline α-chitin fibres in chitin–protein planes that are stacked in a twisted ‘plywood’ structure [55]. Taken together, we propose that the configuration diversity from varied GH18 chitinase sizes, associated CBM5 domains with likely *α*-chitin affinity and further capability caused by the presence (or absence) of linkers between catalytic sites and binding domains have adaptive advantages for saprotrophy within the naturally heterologous organic matrix in which the primarily *α*-chitin substrate is intertwined.

The presence of chitin-binding CBM5 domains is a distinguishing feature of the chitinophilic chytrid genomes in this study. Bacteria-like chitin-binding CBM5 domains were in the *R. globosum* JEL800 genome and other chitinophilic chytrid genomes, suggesting that HGT has taken place, potentially from chitinophilic bacteria to chitinophilic chytrids. The acquisition of chitin-binding capability likely improved the functional efficiency of chytrid chitinases to degrade extracellular chitin-based substrates and therefore has clear fitness advantages. As we propose here for aquatic chitinophilic chytrids, anaerobic gut Neocallimastigomycetes HGT occurred from functionally similar bacteria that likely co-occur in the same niche i.e. anaerobes in herbivore guts that degrade plant-derived polysaccharides and is especially associated with CAZymes and extracellular polysaccharide degradation [56]. Murphy *et al.* [56] proposed that HGT, including the acquired ability to degrade plant-derived polysaccharides, was a key feature of the expansion of the Neocallimastigomycetes, as we propose here with the evolution of the chitinophilic Chytridiales and Polychytriales. Future work is needed on HGT from chitinophilic bacteria to chitinophilic chytrids, including the possibility of co-transferred genes. Here we focused on *R. globosom* JEL800, future studies could also consider HGT across all the currently known chitinophilic chytrids (figure 5), including comparison between the chitinophilic Chytriomycetaceae and the Polychytriales chitinophilic *Polychytrium aggregatum*.

Our study has discovered mechanisms that likely underpin and have supported the specialism of chitin-based saprotrophy in some chytrids. We propose that the complementary expansion of GH18, GH19 and GH20 genes, including potentially from cell wall remodelling functions, alongside niche-specific novel gene transfer of chitin-binding CBM5 domains, potentially from functionally similar bacteria, facilitated chitin saptrophy evolution in chytrids. Expansion of chitin degradation encoding genes and acquisition of CBM5 domains and linkers allowed the diversification of secreted chitinases that may facilitate utilization of chitin within the complex arthropod exoskeleton matrix and, along with substrate-dependent zoospore behavioural traits, determined the widespread and successful exploitation of the chitin-based particle niche in aquatic ecosystems.

## Data Availability

Data are provided as electronic supplementary material. Transcriptome data are available via the European Nucleotide Archive (ENA) PRJNA1076503. Supplementary material is available online [57].
